# Anti-inflammatory effects of Sanguisorbae Radix water extract on the suppression of mast cell degranulation and STAT-1/Jak-2 activation in BMMCs and HaCaT keratinocytes

**DOI:** 10.1186/s12906-016-1317-4

**Published:** 2016-09-06

**Authors:** Ju-Hye Yang, Jae-Myung Yoo, Won-Kyung Cho, Jin Yeul Ma

**Affiliations:** Korean Medicine (KM) Application Center, Korea Institute of Oriental Medicine, 70 Cheomdan-ro, Dong-gu Daegu, 41062 Republic of Korea

**Keywords:** Sanguisorbae Radix, Mast cells, Degranulation, Keratinocytes, Chemokines

## Abstract

**Background:**

Sanguisorbae Radix (SR) is a well-known herbal medicine used to treat inflammatory disease and skin burns in Asia. In addition, it is used to treat many types of allergic skin diseases, including urticaria, eczema, and allergic dermatitis. SR has been reported to exhibit anti-wrinkle, anti-oxidant, and anti-contact dermatitis bioactivities.

**Methods:**

In this study, we investigated the mechanism underlying the anti-inflammatory effects of SR water extract (WSR) using human keratinocyte (HaCaT) cells and BALB/c mouse bone marrow-derived mast cells (BMMCs). Viability assays were used to evaluate non-cytotoxic concentrations of WSR in both BMMCs and HaCaT cells. To investigate the effect of WSR treatment on the degranulation of IgE/Ag-activated BMMCs, we measured the release of β-hexosaminidase (β-HEX). We determined the production of pro-inflammatory chemokines including thymus and activation regulated chemokine (TARC; CCL17), regulated on activation, normal T-cell expressed and secreted (RANTES; CCL5), macrophage-derived chemokine (MDC; CCL22), and interleukin 8 (IL-8; CXCL8) in stimulated human keratinocytes. The ability of WSR to reduce the expression of pro-inflammatory marker proteins was evaluated by Western blotting in HaCaT cells stimulated with tumor necrosis factor (TNF)-α/interferon (IFN)-γ.

**Result:**

WSR inhibited IgE/Ag-activated mast cell degranulation in BMMCs. Treatment with various concentrations of WSR decreased β-HEX release in a dose-dependent manner with an IC_50_ of 27.5 μg/mL. In keratinocytes, WSR suppressed TNF-α/IFN-γ-induced chemokine production and pro-inflammatory molecules *via* a blockade STAT-1, Jak-2, p38, and JNK activation.

**Conclusions:**

This results demonstrate that WSR inhibits degranulation of IgE/Ag-activated mast cells and inhibits the production of pro-inflammatory chemokines by suppressing the phosphorylation of p38 and JNK in HaCaT cells.

## Background

Atopic dermatitis (AD) is an obstinate chronic inflammatory skin disease that progresses through the activation of inflammatory cells such as T cells, monocytes, macrophages, eosinophils, keratinocytes, and mast cells. AD causes epidermal thickness with cutaneous hypersensitivity associated with increased serum immunoglobulin E (IgE) levels and infiltration of inflammatory cell types including mast cells and eosinophils [1–3].

Mast cells are involved in various allergy and inflammatory responses due to the aggregation of the high-affinity IgE receptor (FcεRI) on their surfaces. In recent years, there have been reports that mast cells play an important role in the induction of AD-like skin based on their production of pro-inflammatory cytokines and chemokines. An association between mast cell activation and AD has been suggested by the increase in mast cell number and mast cell activation in AD lesions [2]. In addition, mast cells produce inflammatory mediators such as prostaglandin D_2_ (PGD_2_), and induce eosinophil chemotaxis at inflammatory sites and skin inflammation in AD [3].

Keratinocytes have been shown to be relevant to inflammatory skin responses based on their production of pro-inflammatory chemokines. Stimulation of keratinocytes with TNF-α and IFN-γ induces the expression of pro-inflammatory chemokines including thymus and activation regulated chemokine (TARC; CCL17), regulated on activation, normal T-cell expressed and secreted (RANTES; CCL5), macrophage-derived chemokine (MDC; CCL22) and interleukin 8 (IL-8; CXCL8) [4, 5].

Sanguisorbae Radix (SR) is a well-known herbal medicine that is used to treat inflammatory disease and burns in Asia. In addition, it is used to treat many types of allergic skin diseases, including urticaria, eczema, and AD. SR has been reported to exhibit anti-wrinkle, anti-oxidant, and anti-contact dermatitis bioactivities. However, the mechanism of action of SR in AD is poorly understood. In this study, we investigated the mechanism underlying the anti-inflammatory effects of SR water extract (WSR) using human keratinocyte (HaCaT) cells and mouse bone marrow-derived mast cells (BMMCs) to determine the best treatment method of inflammatory skin diseases such as AD.

## Methods

### Cell culture and reagents

BMMCs isolated from male BALB/c mice (*n* = 1) were cultured for up to 10 weeks in RPMI-1640 media containing 2 mM L-glutamine, 0.1 mM nonessential amino acids, antibiotics, and 10 % fetal bovine serum (FBS), with 20 % pokeweed mitogen-stimulated spleen condition medium (PWM-SCM) as a source of interleukin-3 (IL-3). After 3 weeks, greater than 98 % of the cells were verified as BMMCs according to a previously described procedure [6].

Male BALB/c mice (5 weeks old) were purchased from Samtako BioKorea (Osan, Korea). Mice were observed every day for one week during quarantine and acclimation. All animals were maintained under standard conditions of temperature (22.5 ± 0.5 °C), humidity (42.6 ± 1.7 %), 12 h lighting (8:00 AM–8:00 PM, 290 lx), ventilation (10 –15 times per hour), and diet (Teklad Global Diets, Harlan Laboratories Inc., USA). This study was conducted according to the guidelines listed in the Pharmaceutical Affairs Act of Korea Food and Drug Association (KFDA) and approved by the Animal Care and Use Committee of the Korea Institute of Oriental Medicine (KIOM, Daejeon, Korea; reference number #14–035) and performed according to the guidelines of the Animal Care and Use Committee at KIOM.

The HaCaT cell line was maintained in Dulbecco’s Modified Eagle’s Medium (DMEM) supplemented with 10 % FBS and antibiotics (100 U/mL penicillin and 100 μg/mL streptomycin) at 37 °C in a humidified 5 % CO_2_ incubator.

The ingredients of the complete cell culture medium, FBS, and antibiotics were purchased from Lonza (Basel, Switzerland). Recombinant human TNF-α, IFN-γ, and enzyme-linked immunosorbent assay (ELISA) kits to measure RANTES and TARC were purchased from BioLegend (San Diego, CA, USA). Cell counting kits (CCKs) for the cell cytotoxicity assay were purchased from Dojindo Molecular Technologies (Kumamoto, Japan). β-Hexosaminidase (p-nitrophenyl-2-acetamido-2-deoxy-β-D-glucopyranoside; PNP-GluNAc) was purchased from Sigma-Aldrich (St. Louis, MO, USA). The primary and secondary antibodies used for Western blotting were purchased from Cell Signaling Technology (Boston, MA, USA). All other chemicals were of reagent grade. All experiments were performed at least in triplicate.

### Preparation of WSR

Sanguisorbae Radix was obtained from Yeongcheon Oriental Herbal Market (Yeongcheon, Korea). All voucher specimens were deposited into the herbal bank of the KM-application center, Korea Institute of Oriental Medicine (KIOM; Daejeon, Korea) after verification by Professor Ki-Hwan Bae of the college of Pharmacy, Chungnam National University (Daejeon, Korea). To prepare the WSR, dried SR pieces (50.0 g) were placed in 1,000 mL distilled water and then extracted by 3 h of heating at 115 °C (Gyeongseo Extractor Cosmos-600, Inchon, Korea). Following extraction, the solution was filtered using standard testing sieves (150 μm) (Retsch, Haan, Germany) and freeze-dried. The freeze-dried extract powder was dissolved in dimethyl sulfoxide (DMSO) and centrifuged at 14,000 rpm for 10 min. The resulting supernatant was filtered (0.2 μm pore size) and then stored at 4 °C prior to use. The acquisition was 5.3642 g and the yield 10.73 %. The powder of WSR was dissolved in 10 % DMSO solution for all experiments. All experiments were performed at least in triplicate, and contain a control group as a vehicle control group containing 0.1 % DMSO.

### Cell cytotoxicity assay

Cell cytotoxicity was analyzed using a CCK. Cells were seeded onto 96-well plates (2 × 10^5^ cells/well). After 24 h, WSR was added at concentrations of 10, 50, 100, and 200 μg/mL, and the plates were incubated for 24 h at 37 °C in a 5 % CO_2_ incubator. CCK solutions were added to each well and the cells incubated for 1 h. Optical density was measured at 570 nm using an ELISA plate reader (Infinite M200, Tecan, Männedorf, Switzerland).

### β-HEX release assay

β-Hexosaminidase (β-HEX) was quantified by spectrophotometric analysis of the hydrolysis of PNP-GluNAc. For cell stimulation, BMMCs (5 × 10^5^ cells/mL) were sensitized overnight with 100 ng/mL anti-dinitrophenyl (DNP) and then stimulated for 15 min with 25 ng/mL DNP-human serum albumin (HSA). To investigate the effects of WSR, varying concentrations were added 2 h prior to the addition of DNP-HSA. The supernatants were harvested according to a previously described procedure [7].

### Measurement of chemokine production

HaCaT cells (1 × 10^6^ cells/well) were seeded onto 6-well plates. After 18 h, the cells were treated with WSR at concentrations of 1, 10 and 50 μg/mL for 2 h at 37 °C in an atmosphere of 5 % CO_2_. After treatment, TNF-α/IFN-γ (each 10 ng/mL) was added to each well and incubated for a further 24 h. The culture medium was then harvested and the levels of chemokines in the supernatants detected using ELISA kits according to the manufacturer's instructions.

### RNA isolation and real-time reverse transcription-polymerase chain reaction (RT-PCR) analysis

Total RNA was isolated from HaCaT cells using the RNA-Spin total RNA extraction kit (iNtRoN, Daejeon, Korea) according to the manufacturer’s instructions. Reverse transcription was carried out in a 20 μl reaction with 1 μg of total RNA transformed into cDNA using AccuPower CycleScript RT premix (Bioneer). For measurements of TARC, RANTES, MDC, IL-8 and β-actin mRNA, the PCR conditions were as follows: 12 cycles of primer annealing at 25 °C for 30 s, cDNA synthesis at 45 °C for 4 min, melting of the secondary structure and cDNA synthesis at 55 °C for 30 s, and heat inactivation at 95 °C for 5 min. The PCR-amplified primers used in this study are described in previously described [5]. Gene expression was quantified by real-time PCR using the AccuPower 2× Greenstar qPCR Master (Bioneer) according to the following protocol: pre-denaturation at 95 °C for 10 min, followed by 40 cycles of 95 °C for 10 s, 60 °C for 30 s, and 72 °C for 30 s. Amplifications were carried out using QuantStudio 6 (LifeScience, ABI, USA), The fold change in the expression of the target gene relative to the control was normalized to β-actin using the 2^-ΔΔCt^ method.

### Western blotting

Protein expression was evaluated by Western blotting according to standard procedures. The cells were pre-treated with WSR and stimulated with TNF-α/IFN-γ during incubation for the indicated periods at 37 °C. Cells were then harvested and resuspended in radio-immunoprecipitation assay lysis buffer (Millipore, Bedford, MA, USA) containing a protease and phosphatase inhibitor cocktail (Roche, Basel, Switzerland). After a further round of centrifugation, cell debris was discarded and the protein concentration in the supernatant was determined using Bradford’s reagent. Equal amounts of protein were subjected to sodium dodecyl sulfate-polyacrylamide gel electrophoresis (SDS-PAGE). The separated proteins were transferred onto a nitrocellulose membrane (Millipore, MA, USA) followed by blocking with 3 % bovine serum albumin (BSA) in Tris-buffered saline containing 0.1 % Tween 20. The membrane was incubated first with the primary antibodies at 4 °C overnight followed with horseradish-peroxidase (HRP)-conjugated secondary antibodies for 2 h at room temperature. Specific proteins were detected using Clarity™ West ECL Substrate (Bio-Rad Laboratories, CA, USA).

### Statistical analysis

Data were analyzed using GraphPad Prism software (ver. 5.0 GraphPad Software, San Diego, CA, USA). Results are expressed as the mean ± standard error of the mean (SEM) and were evaluated using Student's t-test or analysis of variance (ANOVA). A *p* value less than 0.05 was considered statistically significant.

## Results

### Effects of WSR on BMMC and HaCaT cell cytotoxicity

**Fig. 1:**
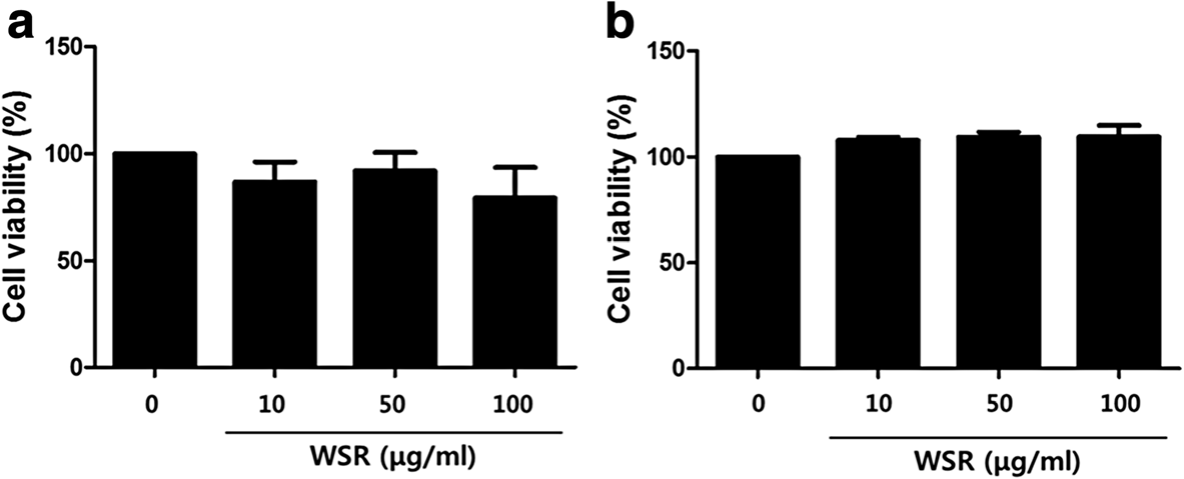
Cytotoxic effects of WSR on HaCaT cells and BMMCs. Cell viability was determined using a CCK assay. Each cells were seeded into 96-well plates and treated with various concentrations of WSR for 24 h. **a** Cytotoxic effects of WSR on HaCaT cells. **b** Cytotoxic effects of WSR on BMMCs. The data are presented as the means ± SEM of three experiments

Cytotoxicity assays were performed to determine the concentration of WSR on mast cells and keratinocyte cells that would not affect cell viability. Each cell type was incubated with different concentrations of WSR (10, 50 and 100 μg/mL) for 18 h and subjected to the CCK-8 assay. A concentration of 50 μg/mL WSR had no significant cytotoxic effect on either BMMCs or HaCaT cells over a 24 h period (Fig. 1). However, WSR at 100 μg/mL had a mild cytotoxic effect on HaCaT cells. Thus, we used WSR at concentrations of 1, 10 and 50 μg/mL for subsequent experiments.

### Effects of WSR on mast cell degranulation

**Fig. 2:**
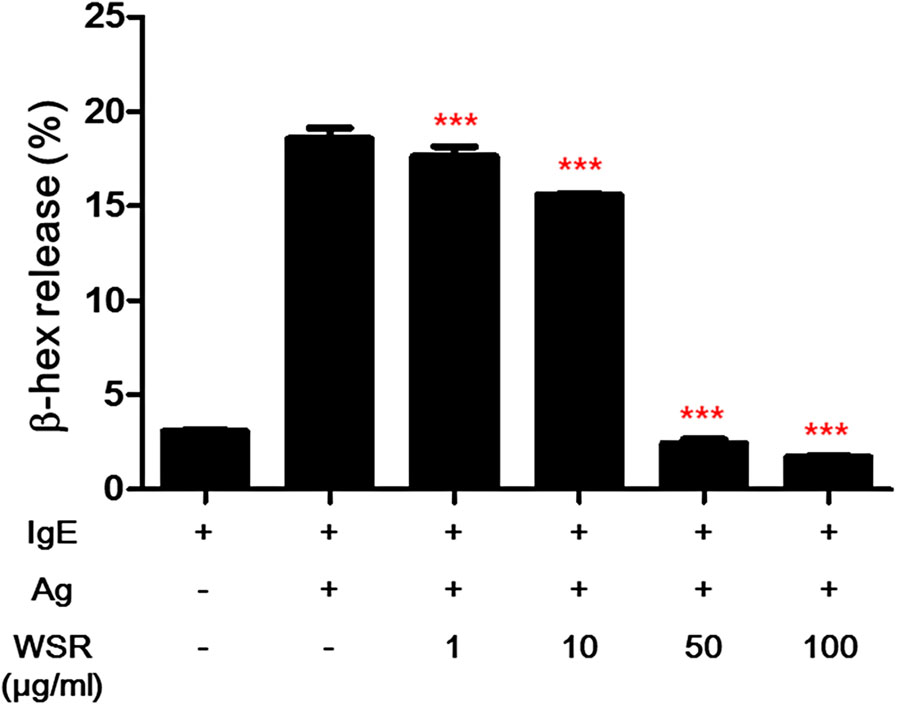
Inhibition of mast cell degranulation by Sanguisorbae Radix water extract. BMMCs were sensitized with IgE (anti-DNP, 100 ng/mL) overnight and then pre-treated with WSR for 2 h. The pre-treated BMMCs were stimulated with Ag (DNP-HSA, 25 ng/mL). β-HEX (a marker of degranulation in mast cells) was measured by spectrophotometric analysis. Data are presented as the mean ± SEM of three experiments. Significance ^***^ *p* < 0.001

Mast cells contain numerous granules and a high-affinity IgE receptor (FcεRI) on their surface. Activated mast cells are degranulated and secrete various pro-inflammatory mediators in their granules such as histamine, TNF-α, and IFN-γ [3]. To investigate the effect of WSR on mast cell degranulation, we measured the release of β-HEX in IgE/Ag-activated BMMCs. As a result, WSR strongly inhibited β-HEX release in a dose-dependent manner (IC_50_; 27.5 μg/mL) (Fig. 2).

### Effects of WSR on TNF-α/IFN-γ-induced pro-inflammatory chemokines production in HaCaT cells

**Fig. 3:**
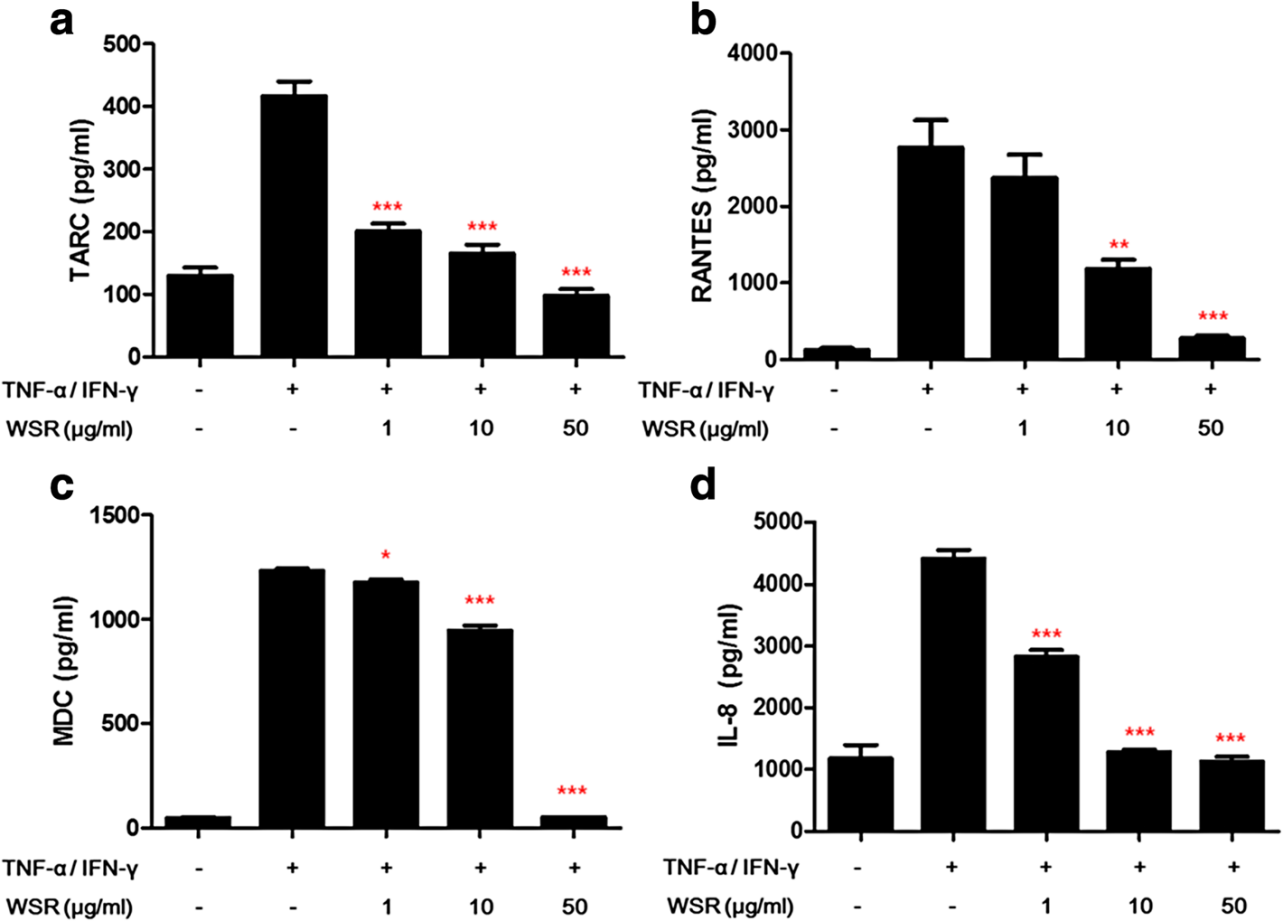
Inhibitory effects of WSR on the TNF-α/IFN-γ-induced production of chemokines in HaCaT cells. The production of TARC (**a**), RANTES (**b**), MDC (**c**), and IL-8 (**d**) was measured in the culture supernatant of TNF-α/IFN-γ-stimulated HaCaT cells. Cells were pre-treated with WSR for 2 h and then stimulated with TNF-α/IFN-γ (each 10 ng/mL) for 24 h. Data are presented as the means ± SEM of three experiments. Significance *p < 0.05, **p < 0.01, ***p < 0.001

To evaluate the anti-inflammatory activity of WSR in human keratinocytes, we measured the TNF-α/IFN-γ-induced production of pro-inflammatory chemokines in HaCaT cells. As shown in Fig. 3, TNF-α/IFN-γ significantly increased the levels of chemokines TARC, RANTES, MDC and IL-8 in HaCaT cells. The results show that WSR inhibited the production of TARC, RANTES, MDC and IL-8 in a dose-dependent manner (each IC_50_; 1, 6, 25 and 25.4 μg/mL, respectively).

### Effects of WSR on TNF-α/IFN-γ-induced pro-inflammatory chemokines expression in HaCaT cells

**Fig. 4:**
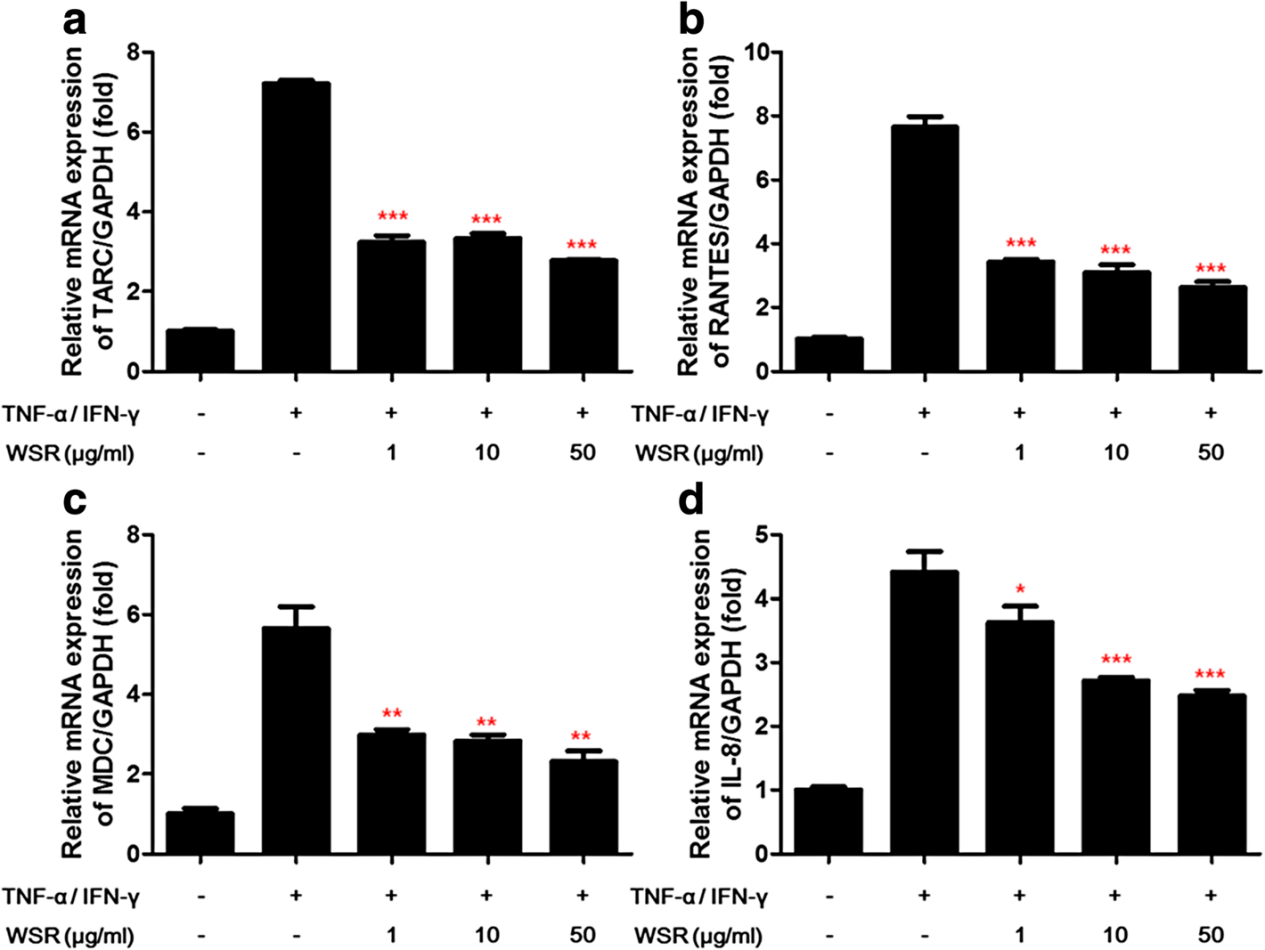
Inhibitory effects of WSR on chemokine expression in TNF-α/IFN-γ-stimulated HaCaT cells. Chemokines are important mediators of the immune response and inflammatory reactions. The exposure of keratinocytes to TNF-α/IFN-γ induces the abnormal expression of chemokines, leading to the infiltration of T cells or leukocytes into inflammatory lesions in the skin [20–22]. We examined the inhibitory effects of WSR on TNF-α/IFN-γ-induced pro-inflammatory chemokine production by HaCaT cells. We assessed the inhibitory effects of WSR on pro-inflammatory chemokine mRNA levels. The expression of TARC, RANTES, MDC, and IL-8 mRNA was then determined using real-time RT-PCR. As shown in Fig. 3, the levels of TARC, RANTES, MDC and IL-8 mRNA were enhanced in TNF-α/IFN-γ-stimulated cells. These increases were dose-dependently inhibited following WSR treatment. Data are presented as the means ± SEM of three experiments. Significance *p < 0.05, **p < 0.01, ***p < 0.001

We investigated the inhibitory effects of WSR on pro-inflammatory chemokine mRNA levels. The expression levels of TARC, RANTES, MDC, and IL-8 genes were determined using real-time RT-PCR. Then, as shown in Fig. 4 stimulation with TNF-α/IFN-γ increased MDC, RANTES, IL-8 and TARC mRNA levels in HaCaT cells. As a result, these were significantly inhibited by WSR treatment in a concentration-dependent manner.

### Effects of WSR on the activation of p38, JNK, and STAT-1/Jak-2 in TNF-α/IFN-γ-stimulated HaCaT cells

**Fig. 5:**
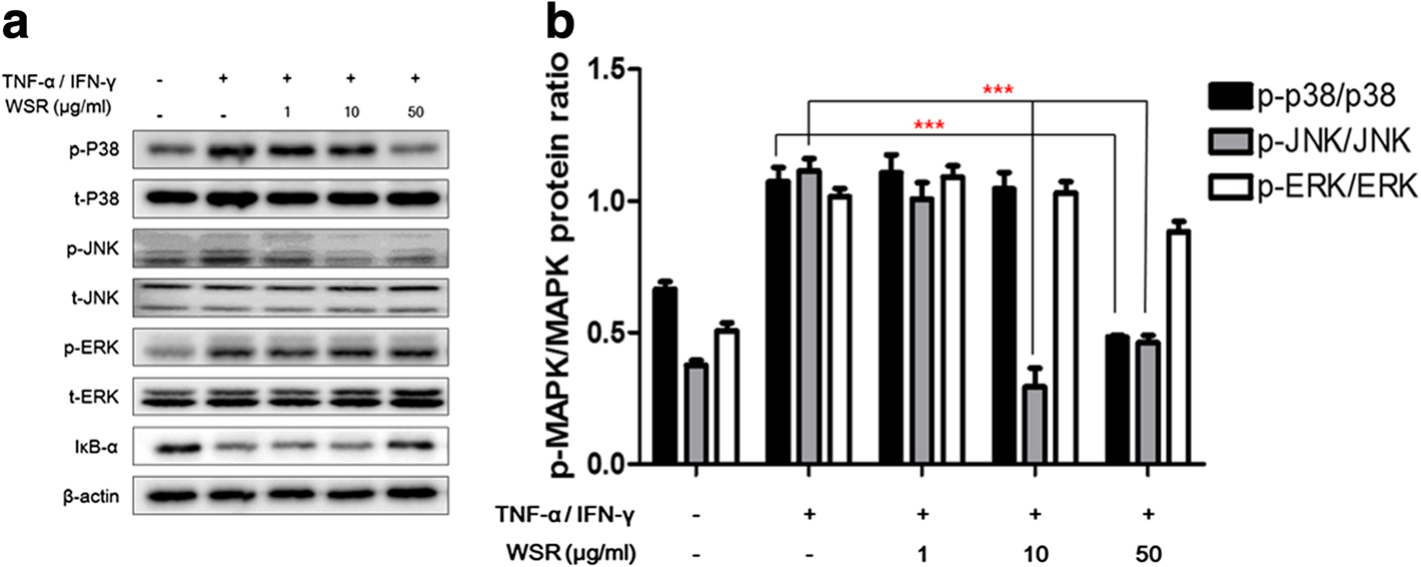
Inhibitory effects of WSR on MAPK phosphorylation in TNF-α/IFN-γ-stimulated HaCaT cells. HaCaT cells were pre-treated with the indicated concentrations of WSR for 2 h and then stimulated with TNF-α/IFN-γ (each 10 ng/mL) for 30 min. The expression or phosphorylation of the indicated proteins was determined in whole-cell lysates by Western blotting using the indicated antibodies. Data are presented as the means ± SEM of three experiments. Significance ***p < 0.001

**Fig. 6:**
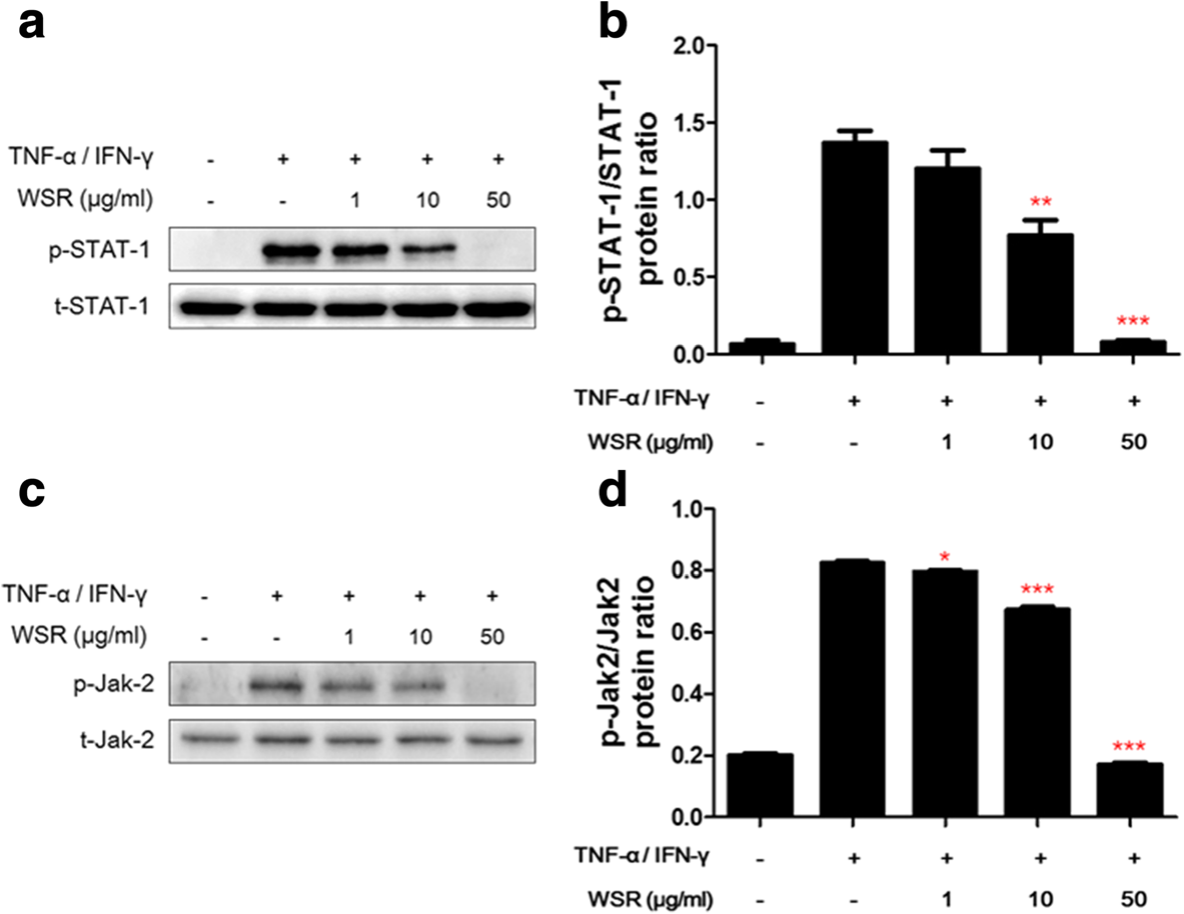
Inhibitory effects of WSR on STAT-1 and Jak-2 phosphorylation in TNF-α/IFN-γ-stimulated HaCaT cells. HaCaT cells were pre-treated with the indicated concentrations of WSR for 2 h and then stimulated with TNF-α/IFN-γ (each 10 ng/mL) for 30 min. **a** The phosphorylation or protein level in whole-cell lysates was determined by Western blotting using antibodies against phospho-STAT-1, STAT, and the band density of p-STAT-1/STA-1. **b** The phosphorylation or level of each protein in whole-cell lysates was determined by Western blotting using antibodies against phosphor-Jak-2, Jak-2, and the band density of p-Jak-2/Jak-2. Data are presented as the means ± SEM of three experiments. Significance *p < 0.05, **p < 0.01, ***p < 0.001. The English in this document has been checked by at least two professional editors, both native speakers of English. For a certificate, please see: http://www.textcheck.com/certificate/6AbvJG

Activation of JNK/ERK/p38 and STAT-1/Jak-2 are closely associated with chemokine production in TNF-α/IFN-γ-stimulated HaCaT cells [8]. Therefore, we investigated whether WSR influences TNF-α/IFN-γ-stimulated HaCaT cells by Western blotting. Cells were stimulated with TNF-α/IFN-γ for a minimum of 5 min and a maximum of 2 h, after which the levels of JNK/ERK/p38 and STAT-1/Jak-2 phosphorylation were determined. The results showed the effects of 50 μg/mL WSR on p38 and JNK activities (Fig. 5). In addition, WSR strongly inhibited the activity of STAT-1 by approximately 53 % at a concentration of 10 μg/mL, and suppressed the activity of Jak-2 by approximately 81 % at a concentration of 50 μg/mL without affecting the total protein level (Fig. 6).

## Discussion

Many studies have reported that high levels of IgE are observed in the serum of AD patients. An increased IgE level in the serum induces the activation of mast cells, which causes an allergic reaction. IgE secretion is an important characteristic of AD, with elevated levels related to disease severity in AD patients [2, 3]. In addition, an association between mast cell activation and AD is suggested by the increase in mast cell counts and activation in AD lesions [9]. IgE binds with a high-affinity receptor for IgE (FcεRI) expressed on the mast cell surface. There are numerous uncertain factors that affect the antigens in AD. The IgE/antigen-bound FcεRI on mast cells leads to cell activation. The activated mast cells degranulate and secrete various pro-inflammatory mediators in their granules such as histamine, TNF-α, and IFN-γ [3, 4, 10]. After the degranulation process, fever and itching is induced in skin lesions by inflammatory mediators that are released from mast cells. Severe itching and the consequential scratching behaviors are factors that deepen in AD.

Keratinocytes play an important role in inflammatory skin disease by producing pro-inflammatory chemokines. Many studies have reported that TNF-α/IFN-γ treatment increases the production of chemokines in HaCaT cells. TNF-α/IFN-γ-stimulation activates several intracellular signaling pathways, including mitogen-activated protein kinases (MAPKs) and STAT-1/Jak-2 [11] [9]. MAPKs and STAT/Jak signaling pathways have been shown to be involved in the regulation of chemokine production in HaCaT cells. These cascades play an important role in immune responses and regulate the inflammatory signaling pathway [9, 12].

Sanguisorbae Radix has been reported that it possesses some beneficial effects such as anti-cancer [13], anti-inflammation [5, 14], anti-oxidation [15], neuroprotection [16, 17] and renal protection [18]. Currently, we have reported the inhibitory action of its ethanol extract on 2, 4-dinitrochlorobenzene (DNCB)-induced AD model [19]. Nonetheless, the inhibitory mechanism of Sanguisorbae Radix in AD responses is still unclarified. Therefore, in present study, we establish how aqueous extract of Sanguisorbae Radix possesses the inhibitory actions against AD responses in both IgE/Ag-activated mast cells and TNF-α/IFN-γ-stimulated keratinocytes.

In present data, when IgE-sensitized BMMCs were preincubated with WSR prior to antigen challenge, WSR dose-dependently inhibited degranulation of the above cells. It suggests that WSR has anti-allergic action by inhibiting degranulation of IgE/Ag-activated mast cells. In addition, regulating activation of TNF-α/IFN-γ-stimulated keratinocytes is another important factor for prevention and treatment of AD. In this study, WSR not only inhibited formation of chemokines such as TARC, RANTES, MDC and IL-8, but also suppressed the activation of p38 and JNK as well as STAT-1/Jak-2 pathway in TNF-α/IFN-γ-stimulated HaCaT cells. It indicates that WSR exerts anti-inflammatory action by suppressing chemokine production in TNF-α/IFN-γ-stimulated keratinocytes.

## Conclusions

This results demonstrate that WSR inhibits degranulation of IgE/Ag-activated mast cells and inhibits the production of pro-inflammatory chemokines by suppressing the phosphorylation of p38 and JNK in HaCaT cells. 

## Abbreviations

AD: Atopic dermatitis
BMMCs: Mouse bone marrow-derived mast cells
FcεRI: High-affinity immunoglobulin E receptor
IFN-γ: Interferon-γ
IL-8: Interleukin 8
MDC: Macrophage-derived chemokine
RANTES: Regulated on activation normal T-cell expressed and secreted
TARC: Thymus and activation regulated chemokine
TNF-α: Tumor necrosis factor-α
WSR: Water extract of Sanguisorbae Radix
β-HEX: β-hexosaminidase
